# Extreme oceanographic forcing and coastal response due to the 2015–2016 El Niño

**DOI:** 10.1038/ncomms14365

**Published:** 2017-02-14

**Authors:** Patrick L. Barnard, Daniel Hoover, David M. Hubbard, Alex Snyder, Bonnie C. Ludka, Jonathan Allan, George M. Kaminsky, Peter Ruggiero, Timu W. Gallien, Laura Gabel, Diana McCandless, Heather M. Weiner, Nicholas Cohn, Dylan L. Anderson, Katherine A. Serafin

**Affiliations:** 1 United States Geological Survey, Pacific Coastal and Marine Science Center, Santa Cruz, California 95060, USA; 2 University of California, Santa Barbara, Marine Science Institute, Santa Barbara, California 93106, USA; 3 Scripps Institution of Oceanography, University of California, San Diego, La Jolla, California 92093, USA; 4 Oregon Department of Geology and Mineral Industries, Coastal Field Office, Newport, Oregon 97365, USA; 5 Washington State Department of Ecology, Coastal Monitoring & Analysis Program, Olympia, Washington 98504, USA; 6 Oregon State University, College of Earth, Ocean, and Atmospheric Sciences, Corvallis, Oregon 97331, USA; 7 University of California, Los Angeles, Civil and Environmental Engineering, Los Angeles, California 90095, USA; 8 Oregon State University, College of Engineering, Corvallis, Oregon 97331, USA

## Abstract

The El Niño-Southern Oscillation is the dominant mode of interannual climate variability across the Pacific Ocean basin, with influence on the global climate. The two end members of the cycle, El Niño and La Niña, force anomalous oceanographic conditions and coastal response along the Pacific margin, exposing many heavily populated regions to increased coastal flooding and erosion hazards. However, a quantitative record of coastal impacts is spatially limited and temporally restricted to only the most recent events. Here we report on the oceanographic forcing and coastal response of the 2015–2016 El Niño, one of the strongest of the last 145 years. We show that winter wave energy equalled or exceeded measured historical maxima across the US West Coast, corresponding to anomalously large beach erosion across the region. Shorelines in many areas retreated beyond previously measured landward extremes, particularly along the sediment-starved California coast.

## Abstract (web summary)

ENSO end members El Niño and La Niña are linked to elevated coastal hazards across the Pacific region. Here, the authors show that the wave conditions and coastal response for the 2015–16 El Niño indicate that it was one of the most significant events of the last 145 years.

The El Niño-Southern Oscillation (ENSO) explains much of the interannual variability in sea surface temperature, sea-level pressure and atmospheric forcing across the equatorial Pacific, affecting global climate patterns^1^ and economies^2^. For example, global economic losses associated with the extreme El Niño of 1982–1983 have been estimated at over US$11.5 billion^3^ (in 2016 dollars), including significant losses along the coast. The extremes of ENSO oscillations, El Niño and La Niña, have been linked to elevated coastal hazards, particularly during boreal winter (December-February) for the Eastern North Pacific (for example, Hawaii, California and the Pacific Northwest (that is, Oregon and Washington^4^,^5^) and Southwestern Pacific (for example, New Zealand^6^ and Australia^7^). El Niño events have also been associated with hazardous coastal conditions in Japan during the boreal fall^8^, greater frequency of tropical cyclone development in the Eastern Pacific^9^ and rotational shifts of embayed beaches in Australia^10^,^11^. With seasonally elevated water levels, higher wave energy and southerly wave directional shifts common during El Niño, the North American west coast has historically experienced severe coastal erosion during El Niño winters, as reported during the 1982–1983, 1997–1998 and 2009–2010 events^4^,^5^,^12^,^13^,^14^,^15^,^16^.

By various metrics, the 2015–2016 El Niño winter was one of the three strongest events in the historical record^17^. For example, in the boreal winter of 2015–2016 the Oceanic Niño Index, a 3-month running mean of sea surface temperatures in the eastern tropical Pacific^18^, reached the highest value in its 66-year history (Fig. 1a). Based on a reconstruction that dates back to 1871 for the multivariate ENSO index^19^, a comprehensive assessment of conditions in the tropical Pacific Ocean^20^—which is significantly correlated with wave energy flux across the Eastern North Pacific^5^—the 2015–2016 winter was only exceeded by the similarly powerful El Niño events of 1982–1983 and 1997–1998 (Fig. 1b). However, a detailed record of coupled oceanographic forcing (that is, waves and water levels) and coastal response during these powerful events is limited primarily to anecdotal reports for the 1982–1983 event^13^, and a few discrete published data sets from the winter of 1997–1998 (refs 12, 14, 21). Further, climate change projections suggest a possible increase in the frequency of extreme El Niño and La Niña events^22^,^23^, which would affect coastal communities across the entire Pacific Basin margin^5^, making it critical to document the forcing and response of historically strong events as a possible proxy for future coastal vulnerability.

Here we provide a detailed assessment of wave conditions, water levels and coastal response during one of the most significant El Niño events of the Industrial Age: the 2015–2016 El Niño. The study analyzes two decades of winter oceanographic forcing across the US West Coast, focusing on the response of 29 beaches along the California, Oregon and Washington coasts, fronting a population of ∼25 million. The region experienced substantial increases in coastal hazards during previous El Niño winters, and has been shown to broadly represent conditions across the Central and Eastern North Pacific^5^,^15^. Both short- and long-term planning needs of coastal communities rely on assessments of the impacts of extreme El Niños due to the temporal scales of coastal hazard vulnerability, ranging from interannual storm hazard fluctuations to multi-decadal wave climate evolution and accelerating sea-level rise.

## Results

### Oceanographic forcing during the 2015–2016 El Niño

**Figure 2:**
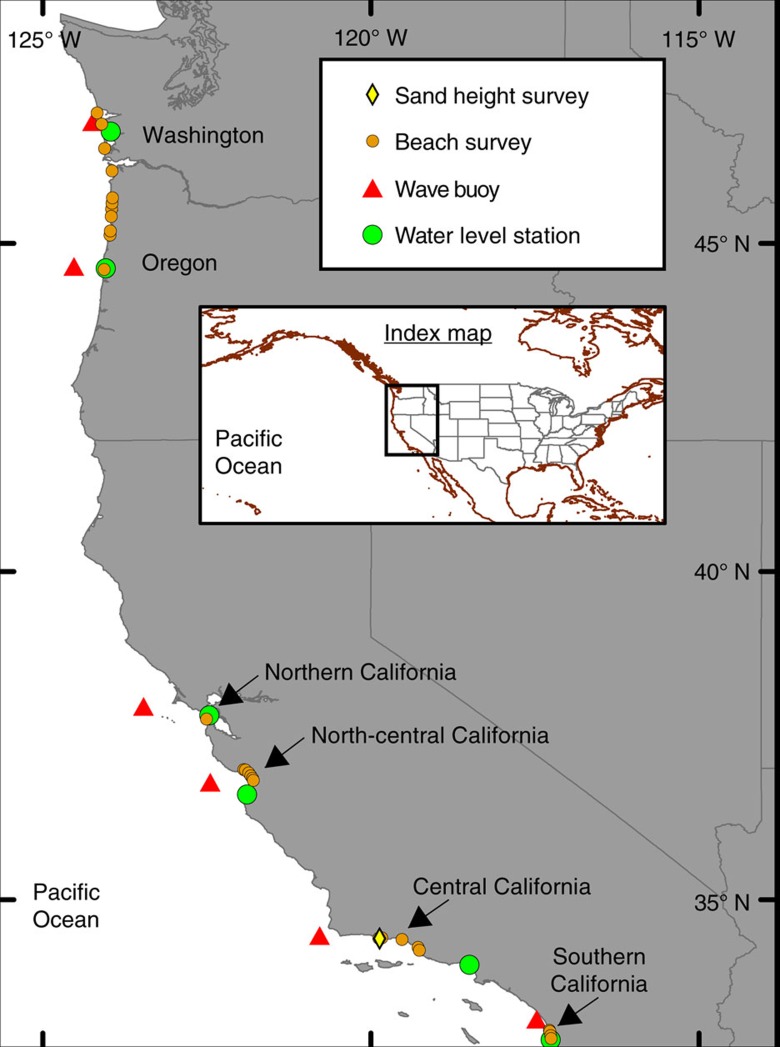
Study area. Locations of the six regions where co-located wave, water-level and beach survey data were analysed. Note that the northernmost beach survey location in Oregon is included in the analysis of Washington (that is, the Columbia River Littoral Cell, for which three of the four locations are in Washington).

The wave climate in the Eastern North Pacific varies seasonally, with larger waves in the fall and winter months driven by the development and passage of extra-tropical cyclones across the mid-latitudes, as well as episodic Eastern Pacific tropical storms in the summer and fall. High pressure dominates in the spring and summer months, with prevailing northwesterly winds and southern hemisphere storms typically resulting in lower wave energy conditions^4^,^24^,^25^. Winter wave energy flux/direction and water-level anomalies were determined from 1997 to 2016 for six wave buoys and six tide gauges, respectively, representing conditions across a 2,000-km section of the west coast of North America, and co-located with beach surveys grouped into six distinct geographic regions (Fig. 2; Supplementary Data 1).

**Figure 3:**
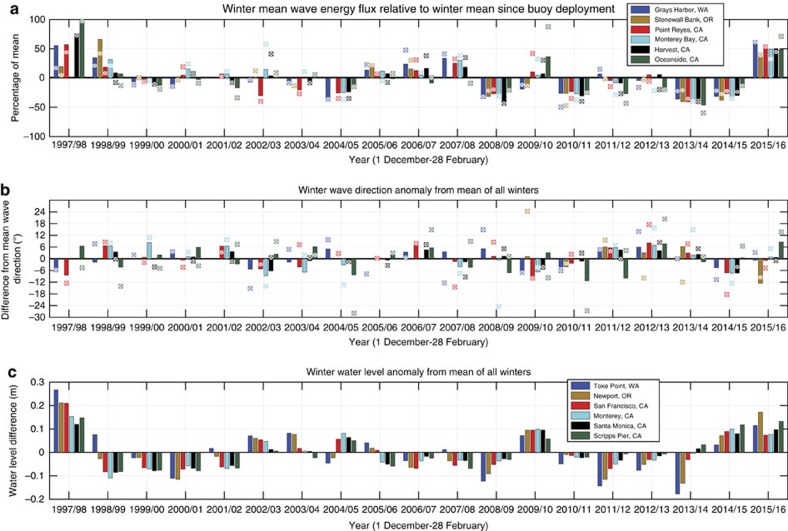
Oceanographic forcing anomalies along the US West Coast. (**a**) Wave energy flux anomalies. Winter (December through February) anomaly (change) in mean wave energy flux relative to the winter mean from 1997–2016. The anomaly of the top 5% (that is, ‘elevated') of the winter wave energy flux relative to the mean of all winters is plotted with squares. See Supplementary Data 2 for the top 0.1, 0.5, 1 and 2% wave energy flux anomalies. (**b**) Wave direction anomalies. Anomaly in winter mean peak wave direction (+ is North, − is South) relative to the overall winter mean. The wave direction anomaly for the top 5% of the winter wave-energy flux measurements from the top panel are plotted with squares (wave data sources: http://cdip.ucsd.edu; http://www.ndbc.noaa.gov/). Note the legend placed in **a** also refers to the buoy locations in **b**. See Supplementary Data 2 for the top 0.1, 0.5, 1 and 2% wave energy flux direction anomalies. (**c**) Water-level anomalies. Anomaly in winter mean water-level relative to the winter mean of all years since 1997 (water-level data source: http://tidesandcurrents.noaa.gov/). The six wave buoy and water-level station measurement locations are listed from north (top) to south (bottom) in the legends, and correspond to each of the six regions used for coastal change analysis (Fig. 2). See Supplementary Data 2 for all the data supporting this figure.

As a key driver of coastal change, mean and elevated (that is, top 5%) wave energy flux (a function of wave height and period, see Methods), were ∼50% above normal averaged across all regions during the 2015–2016 El Niño winter. During the 19 years of analysis, mean wave energy flux was only exceeded by the 1997–1998 El Niño (61% above normal), but elevated wave energy flux in 2015–2016 was the highest on record (Fig. 3a; Supplementary Data 2: note the top 0.1, 0.5, 1 and 2% of wave energy flux is also included in this table, yielding results consistent with the top 5% metric for elevated wave conditions, but with an even greater discrepancy in elevated wave energy flux for the winter of 2015–2016. In the text hereafter, however, we refer exclusively to the top 5% as ‘elevated'). The elevated winter waves of 2015–2016 brought two to four times more wave energy flux than the preceding anomalously low-energy winters of 2013–2014 and 2014–15. Further, one of the most energetic single wave events in the history of the regional wave buoy network struck on 10–11 December 2015, with significant and maximum wave heights off the California and Oregon coasts ranging from 8 to 11 m and 12 to 19 m, respectively^26^.

An unusual aspect of the 2015–2016 oceanographic conditions was the lack of a regionally consistent wave direction anomaly typical of prior El Niño winters^5^. Elevated wave energy flux in particular approached from more southerly angles during the 1997–1998 and 2009–2010 events, ranging from 4° to 13° south of the mean for the California and Washington regions. In contrast, mean and elevated wave energy flux direction in the winter of 2015–2016 was relatively close to the 20-year mean at most sites along the US West Coast, although the Southern California region did experience a marked northerly shift in elevated wave energy flux direction of 18° and 24° relative to the 1997–1998 and 2009–2010 winters, respectively, while Oregon recorded a southerly shift of 10° relative to the mean (Fig. 3b).

Seasonal water-level anomalies averaged 11 cm above the mean across the study area during the winter of 2015–2016, with the highest anomaly (+17 cm) measured on the Oregon coast (Fig. 3c). The anomalies were significantly less than in 1997–1998 across all regions, particularly in Northern California and in the Pacific Northwest (averaged +23 cm in 1997–1998). In California, the water-level anomalies approximated those recorded during the 2009–2010 El Niño and the winter of 2014–2015, where the latter non-El Niño-related water-level anomaly was driven by a high-amplitude upper level ridge that persisted for several years in the Gulf of Alaska, promoting high pressure and unusually high sea surface temperatures^27^ and associated steric effects along the west coast of North America. Wave and water-level patterns calculated over the extended time period of the full fall/winter storm season (October through March) yielded similar results with somewhat muted wave energy flux anomalies (Supplementary Fig. 1; Supplementary Data 2).

### Coastal response during the 2015–2016 El Niño

Beach morphology responds to the seasonal modulation in forcing across the Eastern North Pacific, with beaches tending to build seaward (prograde) during the low wave energy summer months and retreat landward (erode) in the stormier winter months^21^,^28^,^29^. As coastal populations and infrastructure are most susceptible to storm hazards (for example, flooding, cliff failures and structural damage due to elevated water levels and wave attack) when beaches are depleted, we use the relative movement of a representative shoreline contour (a proxy for beach volume change^15^) to assess the magnitude of coastal response and vulnerability.

**Figure 4:**
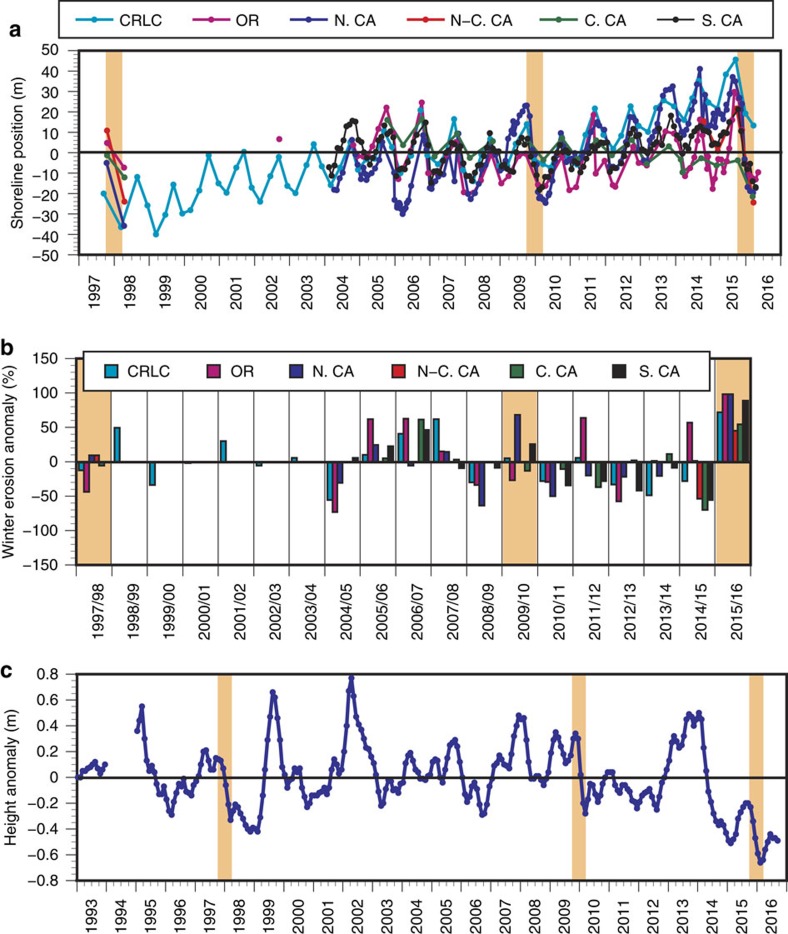
Beach response across the US West Coast. (**a**) Time series of shoreline change. De-meaned shoreline position from each region, assimilating the results from 29 surveyed beaches into six study regions (Fig. 2). For local shoreline proxy information, see Supplementary Data 1. (**b**) Annual winter erosion anomalies. Maximum annual shoreline excursion relative to the mean of all years. See Supplementary Data 2 for the supporting data. (**c**) Twenty-three year record of sand height. Sand height time series from Isla Vista beach in Central California. Areas shaded in orange highlight the El Niño events of 1997–1998, 2009–2010 and 2015–2016. (CRLC, Columbia River littoral cell (that is, Washington and northernmost Oregon); OR, Oregon; N. CA, Northern California; N-C. CA, North-central California; C. CA, Central California; S. CA, Southern California).

Seasonal beach behaviour was assessed for 29 beaches along a ∼2,000 km span of the US West Coast that have been surveyed using aerial Light Detection and Ranging (Lidar), global positioning system-based (GPS) topographic beach surveys with All-Terrain Vehicles and backpacks, and/or discrete measurements of sand levels (Figs 2 and 4; Supplementary Data 1). Temporal survey resolution varies from ∼daily to semi-annual dating as far back as 1993, encompassing the El Niño events of 1997–1998, 2009–2010 and 2015–2016.

Averaged across the six regions of the US West Coast, the winter shoreline retreat of 2015–2016 was the highest on record, with erosion 76% above the normal winter shoreline retreat, 27% higher than any other winter and easily eclipsing the El Niños of 2009–2010 (+12%) and 1997–1998 (−9%) (Fig. 4b; Supplementary Data 2). At the regionally averaged scale, every region except for Central California experienced the highest seasonal shoreline retreat ever measured, and beaches in Central and North-central California recorded the most landward/eroded shoreline positions ever measured. However, it should be noted that the full extent of erosion during the comparably powerful 1997–1998 event probably was not recorded due to two important factors. First, topographic survey coverage in 1997–1998 was not as spatially extensive as in more recent years, with some of the sections of coastline anecdotally most impacted (for example, California) having particularly poor or spotty coverage, or none at all. Second, the Lidar survey utilized to establish the post-El Niño shoreline for many of the California study sites was not collected until April 1998, when beaches were already rapidly recovering, aided by the greater availability of river-supplied sediments from the anomalously high rainfall that winter. This was not the case in 2015–2016 for watersheds adjacent to the California sites where rainfall was significantly below average compared with a typical winter. In recent years, surveys were more frequent throughout the year and/or were conducted during beach minima conditions in the winter. Nevertheless, the coastal erosion of 2015–2016 pushed many beach shorelines beyond recorded historical extremes, including 11 of the 18 beaches surveyed in California. Further, a near-daily time series of sand levels from a site in Central California, shown to significantly represent beach behaviour across that region^30^, reached a 23-year minimum during the 2015–2016 winter, with only marginal recovery through September 2016, which still represented a record low seasonal value (Fig. 4c). The shoreline retreat recorded in 2015–2016 represents a fourfold increase over the prior, mild wave energy winter of 2014–2015 in Southern California, a fivefold increase over the prior winter in Central California, a threefold increase in North-central California and a twofold increase in Northern California and Washington (that is, the Columbia River littoral cell, which includes a beach in northernmost Oregon). Seasonal erosion on Oregon beaches exceeded 2014–2015 levels by a factor of 1.3.

## Discussion

During the winter of 2015–2016, highly elevated winter wave energy flux (∼50% above normal), coupled with seasonally elevated water levels (+11 cm), drove unprecedented levels of winter shoreline retreat (76% above normal), including the most landward shoreline positions measured for the majority of beaches in California since topographic data collection began 20 years ago. The historical significance of this El Niño can be determined by analysing the relatively consistent record of wave energy, water levels and beach behaviour across the study area (available since 1997), and buoy records that date back to the mid-1970s^4^. These historical records, with wave hindcasts that stretch back to the mid-20th century^31^,^32^, and ENSO index time series that date back to 1871 (refs 17, 18, 19) together suggest the 2015–2016 El Niño was one of the most powerful in the past 145 years, similar to 1982–1983 and 1997–1998.

The primary difference in wave energy flux between the most powerful El Niño events of the past two decades (that is, 1997–1998 and 2015–2016) appears related to a latitudinal shift in the primary storm tracks and resulting wave generation location. Elevated wave energy flux during the winter of 2015–2016 exceeded the 1997–1998 event by 29% in Northern California and the Pacific Northwest, including a 44% increase off the coast of Washington. Conversely, higher mean (+37%) and elevated wave energy (+27%) was measured during 1997–1998 for Central and Southern California compared to the 2015–2016 winter. The distinct northerly wave direction anomaly and the smaller elevated wave energy flux anomaly in Southern California during 2015–2016 relative to 1997–1998 are likely related to storm tracks taking a more southerly route during the 1997–1998 El Niño^33^. In 2015–2016, a coincident decrease in precipitation for Southern California compared with Northern California^34^ was the result of a northerly shift in storm tracks relative to 1997–1998. A northerly shift in storm tracks during the El Niño of 1997–1998 compared with 1982–1983 is suggested by precipitation records across California^34^ as well as reports of significant flooding and coastal erosion^13^,^35^, indicating more pronounced impacts from local storms in Southern California during the 1982–1983 winter relative to 1997–1998. This evidence of a progressive northerly migration of storm tracks during El Niño winters along the US West coast is consistent with the observed multi-decadal trend of poleward Hadley cell expansion and, therefore, the location of the sub-tropical jet stream^36^. Measured multi-decadal increases in wave heights for the Pacific Northwest relative to California^4^,^37^,^38^,^39^ is evidence of this broader trend, as is the predicted poleward migration of storm tracks and correlative northerly shift in the focus of extreme wave impacts along the west coast of North America noted in global wave modelling projections for the 21st century^40^,^41^.

While projections of El Niño frequency and magnitude for the 21st century are variable^42^,^43^, one recent study suggests a potential doubling of extreme El Niños^22^, similar to the strength of the 2015–2016 event. Such a trend would result in more significant hazards risk to coastal communities, which would be compounded by anticipated sea-level rise^44^. In addition to providing insight into possible future conditions when extreme El Niños are more frequent, the 2015–2016 El Niño winter may have disrupted the dynamic equilibrium of many US West Coast beaches for years to come, much like the highly anomalous wave activity and coastal response along the Atlantic coast of Europe during the winter of 2013–2014, the most energetic since at least 1948 (ref. 45).

Although erosive conditions were clearly amplified in 2015–2016, large landward shifts in shoreline positions did not translate to pervasively severe erosion of the dunes and bluffs that back the beaches in the Pacific Northwest. This is likely due to the fact that these beaches have generally been accreting since the previous 2009–2010 El Niño, and were in a significantly prograded state in summer 2015 due to the previous two mild winters (Figs 3a and 4a). As a result, increased beach sand volumes moderated the landward erosion resulting from increased wave energy and water levels. Similarly, recent nourishments along several beaches in Southern California also prevented shoreline retreat from reaching landward extremes during the winter of 2015–2016, thereby providing more storm protection for dunes and adjacent coastal infrastructure^46^. Based on this recent behaviour, such naturally or artificially sediment-rich coastal settings are likely to be more resilient to future storm impacts.

The potential for even more extreme coastal erosion during the 2015–2016 El Niño was also moderated by the earlier onset of peak annual high tides and the seasonal water-level anomaly associated with El Niño. The fall 2015 peaks in California, for example, were significantly earlier than the winter peaks that occurred during the El Niños of 1982–1983 and 1997–1998, thereby reducing the probability for the coincident arrival of the largest waves and water levels during the 2015–2016 event^47^.

While natural or artificial increases in beach volumes may reduce erosion-related hazard risk during extreme El Niños at some beaches, hazard risk on many US West Coast beaches may be worsened by historical and possible future reductions in watershed sediment supply to beaches. Even with major reductions in the coastal sediment supply due to dam construction, which has reduced pre-historical riverine sediment supply by 50% in Southern California^48^ and by ∼80% down the Columbia River in the Pacific Northwest^49^, coastal watersheds remain an important source of sand for many US West Coast beaches^50^,^51^. However, 21st century climate projections clearly suggest a significantly warmer climate for California, coupled with precipitation changes that range from negligible to a 26% reduction, with the most severe potential temperature increases and precipitation decreases tied to the upper end emissions scenarios^52^,^53^, mirroring current trajectories^54^. Along with the historical trend of declining sediment supply, these 21st century climate projections would promote less runoff and reduced fluvial discharge rates^55^, likely further reducing the coastal sediment supply. In addition, the risk of extended drought in the Southwest United States is expected to increase significantly in the coming decades^56^, which, if punctuated by the predicted more frequent extreme El Niño events^22^, could increase coastal hazard threats. Reduced fluvial discharge would cause sand supply to beaches to be particularly depleted in the years leading up to these energetic winters, and the resulting narrower pre-El Niño beaches would provide even less protection than normal from increased El Niño wave attack.

In California, the 2015–2016 El Niño serves as proxy for this potential trend: a multi-year drought^57^ limiting the coastal sediment supply, followed by an extreme El Niño event with accompanying elevated waves and water levels that severely eroded beaches across the region. The 2015–2016 El Niño impacts were particularly acute in Southern and Central California due to the preceding drought combined with unusually low (∼50% below normal) winter precipitation^34^,^58^, which not only heightened coastal erosion but is also limiting subsequent beach recovery. This phenomena is clearly observed in the sand height time series from Central California (Fig. 4c), which shows a sharp decrease coincident with the onset of the drought in 2013, followed by record low sand heights in response to the 2015–2016 El Niño event. Record low sand levels have persisted in this location through September 2016. More modest impacts to sediment supply coupled with mild wave energy winters preceding the event resulted in Pacific Northwest beaches being relatively resilient to the 2015–2016 El Niño.

Water levels anomalies of 7–17 cm above normal were measured across the US West Coast during the El Niño winter of 2015–2016, similar to anticipated global mean sea-level increases expected within the next few decades^44^. Therefore, the 2015–2016 El Niño also provides an indication of future background coastal water-level conditions and the associated beach hazards that will become more common during typical winters. The added potential for severe flooding and erosion will be compounded during El Niño winters with higher wave energy and seasonally elevated water levels, posing increasing threats to coastal populations across the US West Coast and beyond.

## Methods

### Coastal change calculations

Coastal change data sets collected between 1993 and 2016 were compiled from 29 beaches, representing the six regions of Southern California, Central California, North-central California, Northern California, Oregon and Washington (USA; Fig. 2; Supplementary Data 1). Representative shoreline proxies (for example, MSL, MHW and MHHW) were extracted from three primary data sources, aerial Lidar, beach profiles and three-dimensional surface maps, and averaged by region to develop a time series of shoreline evolution. From this time series, the maximum annual winter–spring erosion (*E*) was calculated as the difference between the summer/fall (August–November) maximum and subsequent winter/spring (January–April) minimum to coincide with oceanographic forcing fluctuations. The annual shoreline erosion anomaly for each region was calculated as:


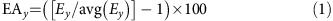


where EA_*y*_ is the erosion anomaly (%), *E*_*y*_ is the erosion in year y and avg*(E*_*y*_) is the mean of this quantity over the entire record. Hence, positive values of the anomaly correspond to erosion larger than the mean.

Mean monthly sand height values from Isla Vista beach, within the Central California region, were calculated from near-daily observations taken at the vertical face of a concrete staircase in the intertidal zone (∼MSL) from 1993 to 2016 (ref. 30). Individual sand height measurements were averaged by calendar month, smoothed using a 3-month running mean, and the average height for each calendar month computed over the entire 23-year record. Monthly height anomalies then were calculated for each month in the record as the difference between the average height in that month and the average for that month over the entire record. Negative values thus correspond to lower than average heights for a given month.

### Wave and water-level statistics

For each of the six study regions, wave (that is, significant wave height, peak wave period and peak wave direction) and water-level data (that is, hourly measured and predicted) were used to assess interannual variability in wave forcing and water-level anomalies to characterize conditions across the US West Coast. Wave energy flux, *F*, was calculated using:


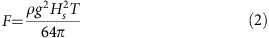


where 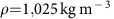 is the density of seawater, 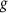, is the acceleration of gravity, *H*_s_ is the significant wave height and *T* is the wave period. Wave directional anomaly was calculated as the number of degrees clockwise (+) or counterclockwise (−) of the peak direction from the average peak direction. Water-level data were gathered from nearby tide stations, which are usually located in semi-enclosed harbours and sheltered from waves. All data were binned into boreal winter (December 1–February 28) and fall/winter (October 1–March 31) averages. A summary of the oceanographic forcing for each region is presented in Supplementary Data 2.

### Code availability

The codes used to generate the results for this project are available upon request from the corresponding author.

### Data availability

All relevant data used in the production of this manuscript are available upon request from the corresponding author.

## Additional information

**How to cite this article:** Barnard, P. L. *et al*. Extreme oceanographic forcing and coastal response due to the 2015–2016 El Niño. *Nat. Commun.* **8,** 14365 doi: 10.1038/ncomms14365 (2017).

**Publisher's note**: Springer Nature remains neutral with regard to jurisdictional claims in published maps and institutional affiliations.

## Supplementary Material

Supplementary Information Supplementary Figure

Supplementary Data 1 Data inventory for study site information.

Supplementary Data 2 Summary of oceanographic forcing (data for Fig. 3) and coastal response (data for Fig. 4). Numbers in bold and highlighted in yellow are the maximum values of the time-series for each region. For wave direction the most negative (southerly shifts) are bolded/highlighted.

Peer Review File

## Figures and Tables

**Figure 1:**
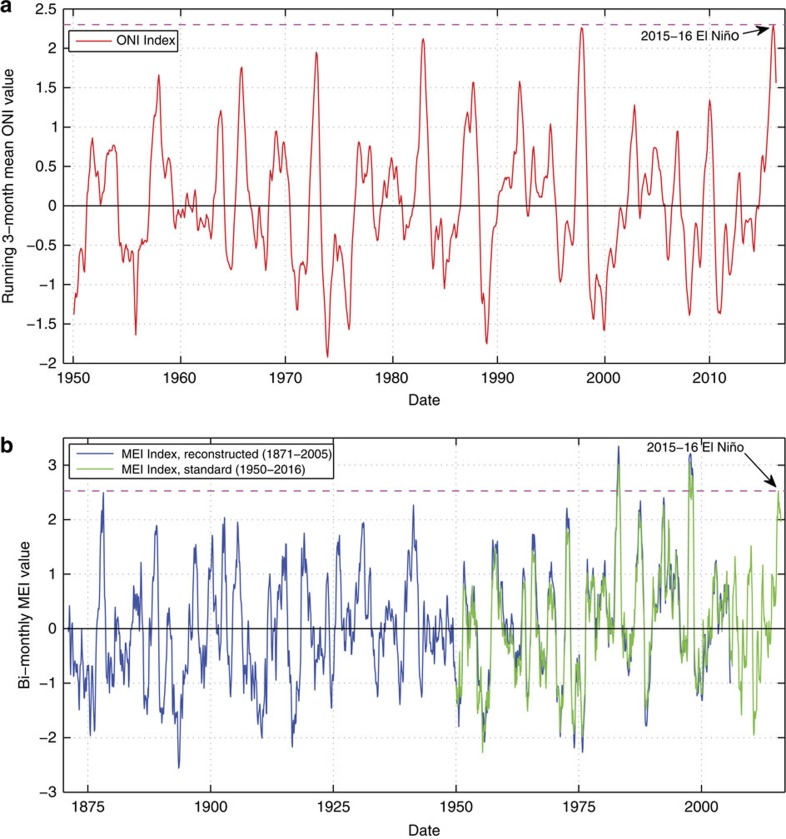
Historical time series of two ENSO indexes. (**a**) Oceanic Niño Index (ONI) from 1950 to 2016. Time series of the ONI tracking the 3-month running-mean of sea surface temperature in the East-central tropical Pacific since the inception of the index in 1950 (ref. 18). The El Niño threshold is reached when the ONI reaches +0.5 or greater for 5 consecutive months. The horizontal pink line marks the maximum ONI index value during the 2015–2016 El Niño (data source: http://www.cpc.ncep.noaa.gov/data/indices/oni.ascii.txt). (**b**) Multivariate ENSO Index (MEI) from 1871 to 2016. Reconstructed time series of the MEI from 1871 to 2005 based on Hadley Centre sea-level pressure and sea surface temperatures^19^ (data source: http://www.esrl.noaa.gov/psd/enso/mei.ext/table.ext.html), and MEI values from 1950 to 2016 based on the six standard observed variables over the tropical Pacific: sea-level pressure, zonal and meridional components of the surface wind, sea surface temperature, surface air temperature and total cloudiness fraction of the sky^20^ (data source: http://www.esrl.noaa.gov/psd/enso/mei/table.html). Bi-monthly averaged MEI values for the reconstructed and standard time series are significantly correlated (*r*^2^=0.94, *P*-value<0.0001) during the overlapping time period (1950–2005) and with minimal bias (*y*=1.0588*x*+0.0839). The horizontal pink line marks the maximum MEI index value during the 2015–2016 El Niño.
