# Supplementary material for: Extreme oceanographic forcing and coastal response due to the 2015–2016 El Niño
Source: Nat Commun. 2017 Feb 14;8:14365. doi: 10.1038/ncomms14365 (PMC5316878; doi:10.1038/ncomms14365)
Supplement: Supplementary Information — Supplementary Figure [file ncomms14365-s1.pdf]

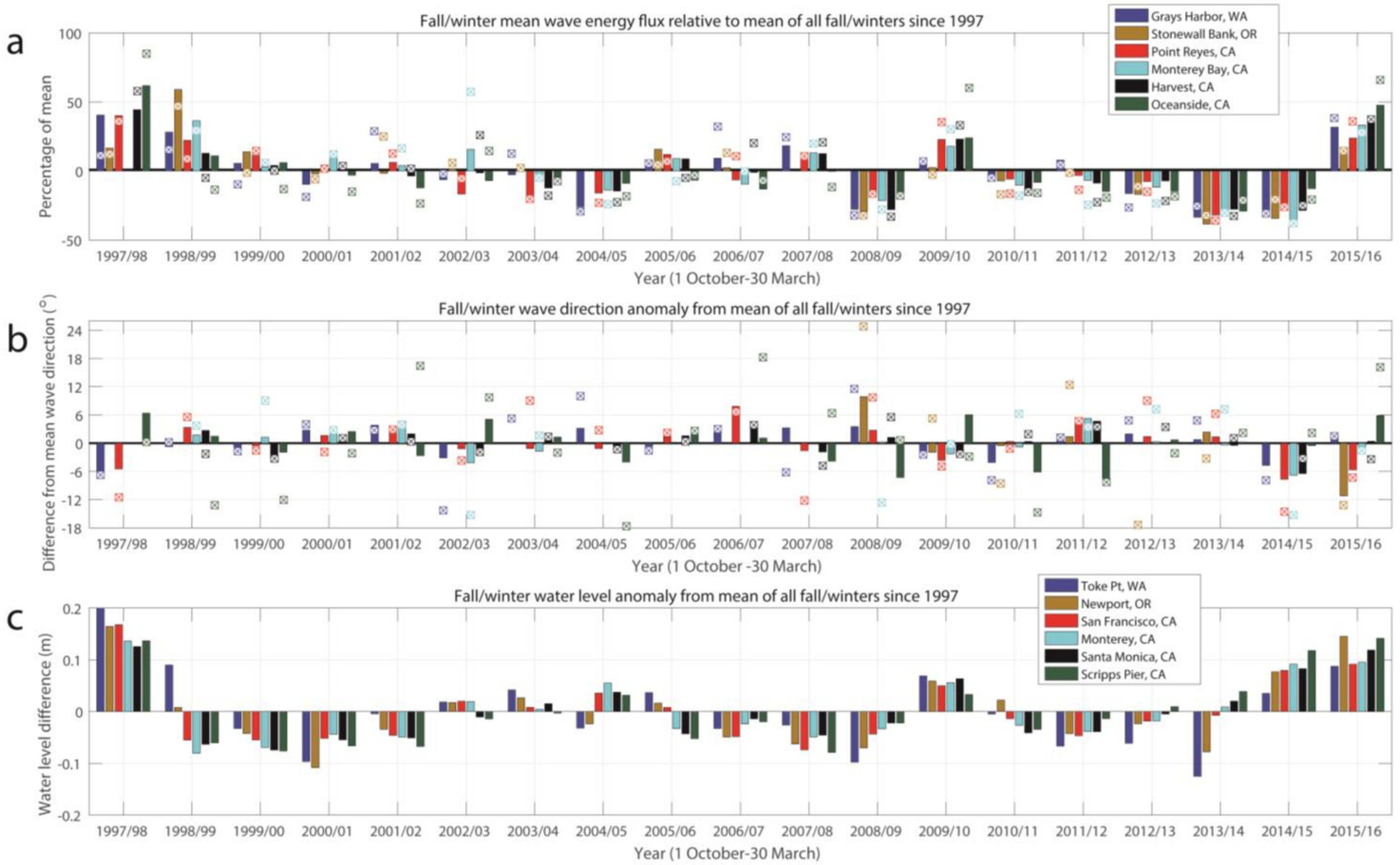

Supplementary Figure 1. Oceanographic forcing anomalies along the U.S West Coast. a) Fall/winter (October through March) anomaly (change) in mean wave-energy flux relative to the mean from 1997-2016. The anomaly of the top 5% (i.e., ‘elevated’) of the fall/winter wave-energy flux relative to the mean of all years is plotted with squares. See Supplementary Data 2 for the top 0.1, 0.5, 1 and 2% wave energy flux anomalies. **b)** Anomaly in fall/winter mean peak wave direction (+ is North, − is South) relative to the overall mean. The wave direction anomaly for the top 5% of the fall/winter wave-energy flux measurements from the top panel are plotted with squares. Note the legend placed in panel ‘a’ also refers to the buoy locations in panel ‘b’. See Supplementary Data 2 for the top 0.1, 0.5, 1 and 2% wave energy flux direction anomalies. **c)** Anomaly in fall/winter mean water level relative to the mean of all years since 1997. The six wave buoy and water level station measurement locations are listed from north (top) to south (bottom) in the legends, and correspond to each of the six regions used for coastal change analysis (see Fig. 2). See Supplementary Data 2 for all the data supporting this figure.
