# Supplementary material for: Extreme oceanographic forcing and coastal response due to the 2015–2016 El Niño
Source: Nat Commun. 2017 Feb 14;8:14365. doi: 10.1038/ncomms14365 (PMC5316878; doi:10.1038/ncomms14365)
Supplement: Peer Review File [file ncomms14365-s4.pdf]

Reviewers' comments:

Reviewer #1 (Remarks to the Author):

Detailed comments are provided in the attached document.

Summary of the key results
* As in the abstract - I find the term 'historical' somewhat misleading

Originality and interest: if not novel, please give references
* The paper contains original and highly relevant material

Data & methodology: validity of approach, quality of data, quality of presentation
* The data are what they are - the paper contains all data available on the topic.

Appropriate use of statistics and treatment of uncertainties
* I do not find the conclusions fully supported by the data. The fact that the more severe El Nino of 1997-1998 caused less than average seasonal change; the Mallorca site which shows more pronounced change in the years 2013-2014; the strong long-term trends at some of the sites; and the influence of antecedent beach state on the seasonal response - these are all factors that seem to have been overlooked and do not support the main conclusion of the paper.

Conclusions: robustness, validity, reliability
* See previous comment.

Suggested improvements: experiments, data for possible revision
* Not sure how to improve significantly. It will have to be a more robust analysis, interpretation an qualification of the results, but that will undoubtedly weaken the (overstated) conclusion.

References: appropriate credit to previous work?
* Appropriate

Clarity and context: lucidity of abstract/summary, appropriateness of abstract, introduction and conclusions
* See above

Review of paper 'Historically significant oceanographic forcing and coastal response due to the 2015-16 El Niño' for *Nature Communications*

I have a real issue with the word 'historically' in both the title and the abstract, which I believe are somewhat misleading. It suggests that the record of oceanographic forcing and coastal response goes back a long time, whereas in fact it only a goes back two decades. The El Niño of 2015-2016 was one of the strongest in the last 150 years (actually, it would be more correctly to say it was the third strongest since the last 150 years; the other two stronger El Niño's were 1982-83 and 1997-98), but the historical records of oceanographic forcing (waves) and response do not stretch back that far. So, what the paper actually demonstrates is that the period 1998-2016, the year 2015-2016, which represents the strongest El Niño over this 20 year period, was characterised by the strongest wave conditions and the most extensive coastal responses.

I have two comments related to the data on beach responses and the implications:

1) The 'strength' and 'direction' of the coastal response is based on comparing the magnitude of the seasonal response with the average seasonal response. Thus, the antecedent conditions are actually very important. Since the preceding winters of 2013-14 and 2014-15 were characterised by 'anomalously low-energy levels' (lines 83-84) it is perhaps not surprising that the winter of 2015-2016 had such a significant impact. Perhaps more telling is the fact that the beaches in Central and North-Central California recorded the most landward/eroded shoreline position in history (again, for the last 20 years). I am deducing that this was therefore not the case at the other sites (confirmed by Figure 4), so the key result relates therefore to the seasonal response, which is as much a function of the storm conditions and the antecedent beach state.
2) Lines 122-124 contain an interesting statement (my bold underlining): 'Averaged across the six regions of the U.S. West Coast, the winter beach erosion of 2015-16 was the highest on record, with erosion 76% above the normal winter shoreline retreat, 27% higher than any other winter, and easily eclipsing the El Niños of 2009-10 (+12%) <u>**1997-98 (-9 %)**</u>'. What it indicates is that the El Nino of 1997-1998, which was stronger than the 2015-2016 El Niño, actually generated coastal responses that were less than average. So, one could argue here that El Niño's have a moderating effect on seasonal beach response.

The Section 'Historical significance of the 2015-16 El Niño' is largely a repeat of the previous two sections (especially the first paragraph). There is some 'new' information that would be better incorporated in the two previous sections.

Lines 173-181 argue that the upper beach and dune response was quite limited during the 2015-16 winter because the beaches were in a relatively healthy state and widespread beach nourishment had taken place over the previous years. But what then explains the below average seasonal response to the stronger El Niño winter of 1997-1998?

Lines 183-198 are somewhat off-topic and rather speculative. It all depends on how important the fluvial sediment supply is for maintaining the beaches, and that really is a different topic/paper.

Lines 208-213 make reference to the sea-level anomaly and how this can be a proxy for sea-level rise. The sea-level anomaly for 1997-1998 far exceeded the 2015-2016 anomaly, but the coastal response during 1997-1998 was less than average. Again, this needs explanation.

The beach change figure 4 contains some rather puzzling results that deserve explanation. Firstly, for several sites there is a significant beach widening trend over the last 20 years – it seems these beaches have significantly decreased their vulnerability. Secondly, the Camino Mallorca site shows the largest reduction in beach level occurred in the years before 2015-2016, directly contradicting the main conclusion of this paper. Thirdly, it is unclear what shoreline is plotted in this figure (MSL, MHW, MHHW).

Reviewer #2 (Remarks to the Author):

This is an important contribution that not only puts the significant of the 2015-16 ENSO event into a longer term historical perspective, but also provides important perspective on what the oceanographic conditions and shoreline responses that were experienced during this winter may mean for the future of shorelines/coastlines of the west coast states. The paper brings together very comprehensive data sets that span an important period of time but also a significantly large geographic area, which is unique relative to past shorter term or smaller areal descriptions or analyses. The quality of the date is high, the multiple authorship adds important breadth and geographic coverage, and the paper is a nice integration of both the forcing functions/physical processes and the shoreline responses.
Figures are nicely done, very understandable and present a great compilation of long term wave/water level/shoreline conditions that will be a valuable data source for other workers.
Methods are straightforward and easy to understand and follow.
I am attaching a few specific comments, some other historic data and references that the authors may or may not be aware of. There have been data collected during previous ENSO winters (1983 and 1997-98) that do provide some added perspective on what was observed this 2015-16 winter, but that weren't referenced.
Abstract is informative and provides a useful and brief summary.
References are extensive although I have added a few others that I believe are pertinent to the discussion and should be considered.

REVIEW OF: Historically significant oceanographic forcing and coastal response due to the 2015-16 El Niño by Patrick Barnard, et al.

Lines 55- 56. However, a detailed record of coupled 55 oceanographic forcing (i.e., waves and water levels) and coastal response during these extreme events is limited primarily to anecdotal reports for the 1982-83 event..

There is one published graphic of the coincidence of predicted tidal levels and wave heights during the 1983 ENSO, which shows why this winter was so damaging to coastal California

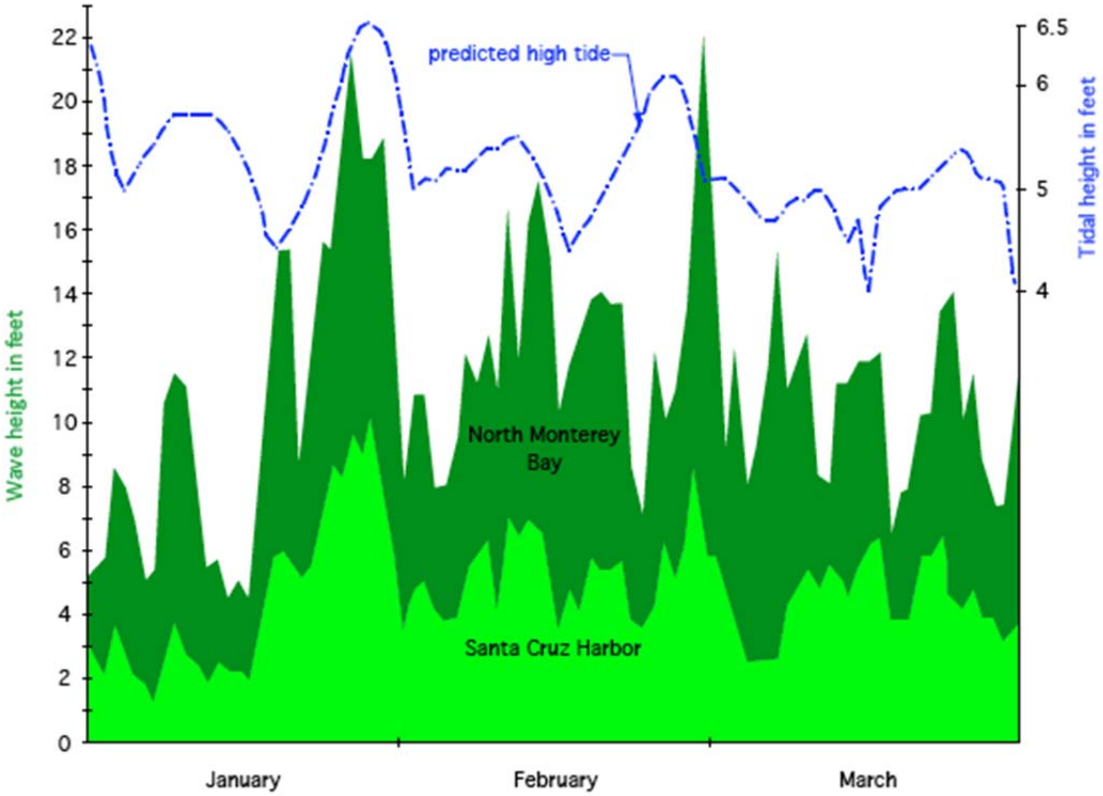

Figure 3.6. The arrival of large storm waves at times of high tides in the first three months of the 1983 El Niño produced millions of dollars in damage along the entire length of California's coast. Santa Cruz Harbor and North Monterey Bay are two different wave gauges. Predicted high tide is the higher high tide for each day.

Line 67. I think convention is to hyphenate **sea-level rise**.

Line 98. Looking at NOAA water level anomalies, unless I am not understanding the use of "highest anomaly" for the 2015-16 water levels, the Monterey, California tide gage recorded an anomaly of 12.9 " or 32 cm anomaly (observed above predicted- although not listed as "verified" when I looked this up initially). See attached plot for January 5, 2016.

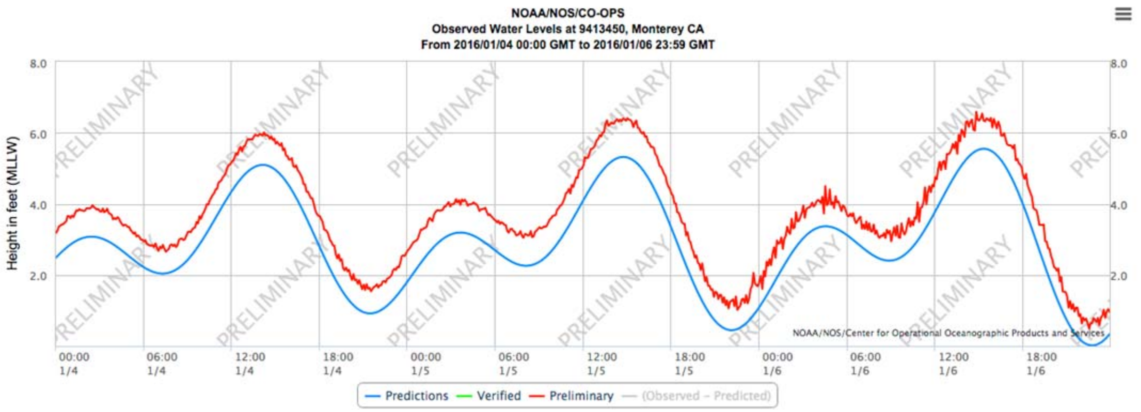

Lines 122-127 These are good data, cover a broad geographic area and put the 2015-16 ENSO winter into recent historical perspective, in terms of coastal impact. There have been historical measurements from prior large ENSO winters but its not clear from these plots that they are included. Do the comparisons include Kristen Brown's MA thesis (while she was working at USGS) on 1997-98 beach profile changes (pre-, mid- and post-1997-98 ENSO- see attached summary) along central California coast, and John Dingler's (also USGS) Monterey Bay beach profiles (USGS El Nino Storms Erode Beaches on Monterey Bay USGS Fact Sheet 026-00, 2000)?

From Kristen Brown MA thesis (UCSC)

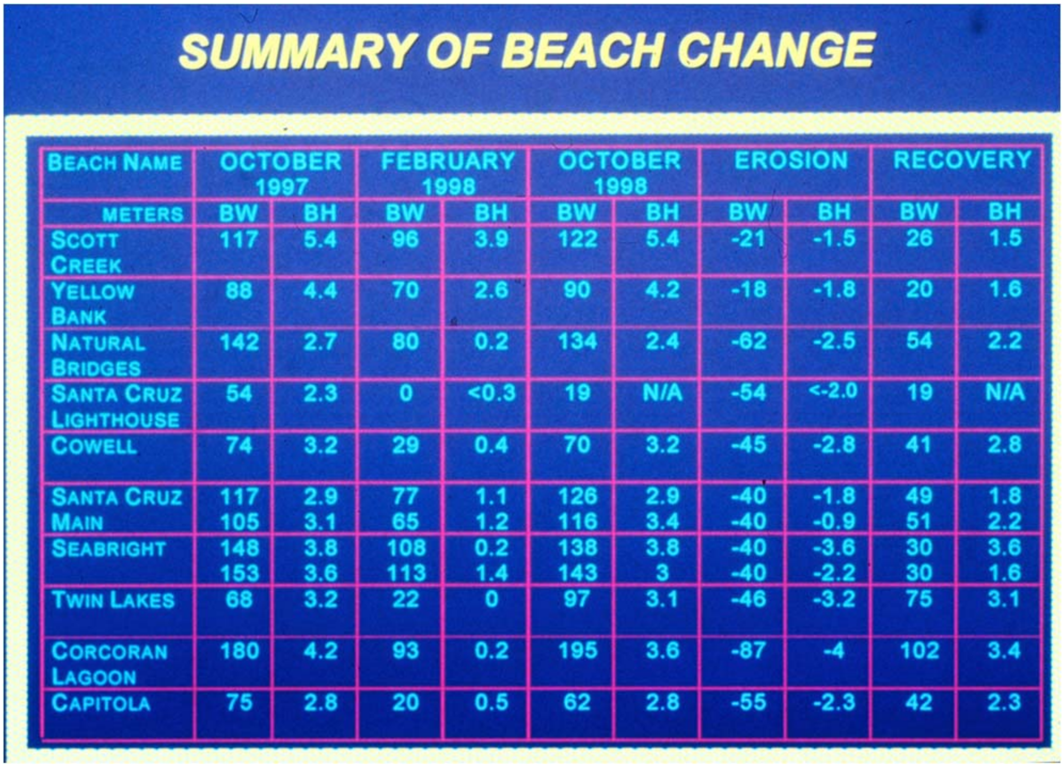

SUMMARY OF BEACH CHANGE

Beach Name	October 1997		February 1998		October 1998		Erosion		Recovery	
Meters	BW	BH	BW	BH	BW	BH	BW	BH	BW	BH
Scott Creek	117	5.4	96	3.9	122	5.4	-21	-1.5	26	1.5
Yellow Bank	88	4.4	70	2.6	90	4.2	-18	-1.8	20	1.6
Natural Bridges	142	2.7	80	0.2	134	2.4	-62	-2.5	54	2.2
Santa Cruz Lighthouse	54	2.3	0	<0.3	19	N/A	-54	<-2.0	19	N/A
Cowell	74	3.2	29	0.4	70	3.2	-45	-2.8	41	2.8
Santa Cruz Main	117	2.9	77	1.1	126	2.9	-40	-1.8	49	1.8
	105	3.1	65	1.2	116	3.4	-40	-0.9	51	2.2
Seabright	148	3.8	108	0.2	138	3.8	-40	-3.6	30	3.6
	153	3.6	113	1.4	143	3	-40	-2.2	30	1.6
Twin Lakes	68	3.2	22	0	97	3.1	-46	-3.2	75	3.1
Corcoran Lagoon	180	4.2	93	0.2	195	3.6	-87	-4	102	3.4
Capitola	75	2.8	20	0.5	62	2.8	-55	-2.3	42	2.3

Lines 144-45- "including the most landward shoreline positions observed for the majority of beaches in California since topographic data collection began twenty years ago".
What is important to keep in perspective, however, is that much of this 20 year period (~1999-2014) was a negative/cool PDO period such that large ENSO events wouldn't have been expected, and that extreme beach erosion or retreat wouldn't have been expected either, in contrast to the previous two decades (1978-1999), a warm/positive PDO cycle.

Line 168: sea-level rise

Lines 169-171- In addition to providing insight into possible future conditions when extreme El 169 Niños are more frequent, the 2015-16 El Niño winter may have disrupted the dynamic equilibrium of many 170 U.S. West Coast beaches for years to come…
The beach surveys done by Kristen Brown as part of her MA thesis with USGS showed severe erosion in 1997-98 along the central coast of California but then full recovery by the next fall.

Lines 192-193: "Along with the historical trend of declining sediment supply, these 21st 192 century climate projections would promote less runoff and reduced fluvial discharge rates, likely further reducing the coastal sediment supply."

Somewhat surprisingly, despite the significant reductions in fluvial sediment supply due to construction of dams and debris basins in southern California, the beaches haven't uniformly or consistently shown reductions in width. (*Orme, A.R., Griggs, G.B., Revell, D.L., Zoulas, J.G., Grandy, C.C., and Koo, J. 2011. Beach changes along the Southern California coast during the twentieth century: A comparison of natural and human forcing factors. Shore and Beach 79(4): 38-50)*

Reviewer #3 (Remarks to the Author):

Review of manuscript Barnard et al.

This manuscript presents an overview of the coastal response along the US west coast, from southern California up to the Washington coast, to the 2015-16 El Nino event, which is identified as one of the strongest El Nino events in the historical record.

The study assesses the local physical drivers, sea level anomalies and wave climate, in context with measurements of shoreline position, to identify that the 15-16 erosional event lead to the most landward shoreline positions observed for the majority of the Californian coast since records began.
The study provides a very timely presentation of the coastal response to the recent El Nino event, and I would anticipate would be of interest to the readership of Nature Communications. I have a few comments below which I would recommend the authors consider before the paper is published.

I commend the authors on a nice study.

(abstract) The manuscript is set up describing the wide spread coastal response along the Pacific margin to El Nino events, but then limits analysis and later discussion to only the US west coast, and becomes quite US centric. Given the other work these authors have been involved in, it would be nice to see discussion of implications more broadly, increasing the interest to a much broader readership.

(line 38), gives a figure of US$11.5 billion damages associated with the 82-83 El Nino. Using this figure, which relates to all El Nino damages – a lot of which are associated with industries well away from the coastal fringe (agriculture, or fisheries), is a bit misleading. Is there a figure available on what the damages associated with coastal erosion/shoreline position/flooding are? This would be a more relevant figure to quote.

(line 79) Referring to the 95th percentile as extreme is not appropriate. Hourly data is used, and the 95th percentile corresponds to an average of every 20th measurement (more than once per day) is greater than this value. Some other method of characterising ‘extreme’ waves should be considered.

(line 79-81) sentence needs editing.

(line 95). The wave conditions during the 15/16 El Nino are identified as being atypical relative to prior el nino events.

On lines 157-162, the authors attribute these differences to background trends in wave climate. The analysis of this is somewhat superficial and I think the paper would be substantially strengthened by a more rigorous analysis of this. The counter argument is that by being atypical, they provide a poor model of what may occur during future El Nino events, as is argued in other sections of the manuscript.

(line 143 – and elsewhere in the manuscript). Reference to wave energy, and wave power (wave energy flux) appears to be used interchangeably without consideration through the manuscript. This should be tightened up.

(line 150-151). The wording of ‘strong latitudinal variations’ doesn’t capture what I think the authors are trying to convey – being the differences in latitude of the storms between the two events. As worded, it suggests that during each event, there is similar variations in latitude of the storms. Some editing of the text needed.

Figure 3, and discussion around line 210. It is not clear whether the positive sea level anomalies along the US west coast are a common signal of all El Nino events, or whether the authors are just proposing this positive anomaly provides some indication of a possible future sea-level rise signal. I think the point is lost that the positive anomalies will on top of the background SLR in future, exacerbating potential effects.

Reviewer #1:

I have a real issue with the word 'historically' in both the title and the abstract, which I believe are somewhat misleading. It suggests that the record of oceanographic forcing and coastal response goes back a long time, whereas in fact it only a goes back two decades. The El Niño of 2015-2016 was one of the strongest in the last 150 years (actually, it would be more correctly to say it was the third strongest since the last 150 years; the other two stronger El Niño's were 1982-83 and 1997-98), but the historical records of oceanographic forcing (waves) and response do not stretch back that far. So, what the paper actually demonstrates is that the period 1998-2016, the year 2015-2016, which represents the strongest El Niño over this 20 year period, was characterized by the strongest wave conditions and the most extensive coastal responses.

We understand the reviewer's concern here, as the reliable wave buoy and coastal response data only go back ~20 years. However, it has been shown in other studies (c.f., Barnard, P. L. *et al.* Coastal vulnerability across the Pacific dominated by El Niño/Southern Oscillation. *Nature Geosci.* **8,** 801–807 (2015) AND Harley, M. D., Barnard, P. L. & Turner, I. L., Linkages between climate oscillations and coastal wave variability across the Pacific basin. Coastal Sediments 2015: The Proceedings of the Coastal Sediments 2015 (World Scientific, 2015)) that El Niño indices like MEI are significantly correlated with wave energy flux and coastal response, and therefore by proxy can be used to extend the record, broadly speaking, back to the onset of the climate index record. That being said, to avoid any miscommunication, we have modified the title to "Extreme oceanographic forcing and coastal response due to the 2015-16 El Niño" which simply summarizes the exceptionality of the event. Also, in the abstract, we modified both sentences using the word "historical" to "…wave energy equalled **observed** historical maxima" and "Shorelines in many areas retreated beyond **observed** landward (deleted **historical**) extremes"

The climate index record clearly suggests that it's quite fair to say that the "The El Niño of 2015-16 was one of the strongest in the last 150 years", not just the "third strongest" as the reviewer suggests. The relative strength of a given El Niño is subject to the metric of choice. The NOAA Climate Prediction Center's operational definition of an El Niño is a positive ONI greater than or equal to 0.5^{o}C for five consecutive overlapping three-month seasons (http://www.cpc.ncep.noaa.gov/products/analysis_monitoring/ensostuff/ensoyears.shtml and https://www.climate.gov/news-features/understanding-climate/climate-variability-oceanic-ni%C3%B1o-index). By that definition, the 2015-16 event was THE strongest on record, reaching the highest value ever calculated in December 2015 (see Figure 1, top). However, the recent and reconstructed MEI time-series (Figure 1, bottom) would place the 2015-2016 event as the 3rd strongest behind 1982-83 and 1997-98. Other ENSO related metrics (e.g., SOI) yield similarly mixed results, so it's not clear exactly where 2015-16 ranks historically, other than it consistently ranks in the top 3 along with 1982-83 and 1997-98. Further, a just released study (Huang, B., M. L'Heureux, Z.-Z. Hu, and H.-M. Zhang (2016), Ranking the strongest ENSO events while incorporating SST uncertainty, Geophys. Res. Lett., 43, 9165–9172, doi:10.1002/2016GL070888), now added to the manuscript, further indicates that 1982-83, 1997-98 and 2015-16 are clearly the strongest on record, but that their relative ranking is difficult to determine due to sea surface temperature measurement uncertainty.

I have two comments related to the data on beach responses and the implications:
1) The 'strength' and 'direction' of the coastal response is based on comparing the magnitude of the seasonal response with the average seasonal response. Thus, the antecedent conditions are actually very important. Since the preceding winters of 2013-14 and 2014-15

were characterized by 'anomalously low-energy levels' (lines 83-84) it is perhaps not surprising that the winter of 2015-2016 had such a significant impact. Perhaps more telling is the fact that the beaches in Central and North-Central California recorded the most landward/eroded shoreline position in history (again, for the last 20 years). I am deducing that this was therefore not the case at the other sites (confirmed by Figure 4), so the key result relates therefore to the seasonal response, which is as much a function of the storm conditions and the antecedent beach state.

This is a valid point, and precisely why we have reported both the seasonal change anomaly AND the beach minimum conditions. Based on equilibrium shoreline theory (e.g., Yates, M. L., R. T. Guza, and W. C. O'Reilly (2009), Equilibrium shoreline response: Observations and modeling, J. Geophys. Res., 114, C09014, doi:10.1029/2009JC005359), the more out of equilibrium the beach state is in relation to the wave energy, the more rapidly it can change. Therefore, we would expect a strong erosional response in response to elevated wave energy along an accreted coast (as the reviewer points out in the Pacific Northwest). Nevertheless, the Pacific Northwest region still experienced record wave energy, and likely would have reached beach minima at many sites if the beaches had entered the winter closer to equilibrium. Further, despite the low energy winters preceding the 2015-16 El Niño, many of the beaches in California experienced BOTH record seasonal retreat AND reached historical minimum conditions (> 60%) during the 2015-16 El Niño. We have updated the text accordingly to further clarify this important point the reviewer raises.

2) Lines 122-124 contain an interesting statement (my bold underlining): 'Averaged across the six regions of the U.S. West Coast, the winter beach erosion of 2015-16 was the highest on record, with erosion 76% above the normal winter shoreline retreat, 27% higher than any other winter, and easily eclipsing the El Niños of 2009-10 (+12%) ***1997-98 (-9 %)****'. What it indicates is that the El Nino of 1997-1998, which was stronger than the 2015-2016 El Niño, actually generated coastal responses that were less than average. So, one could argue here that El Niño's have a moderating effect on seasonal beach response.*

Above we explained in large part this unexpected "moderate" behavior of the 1997-98 El Niño. Also, several lines later in the original text we explained that this moderate behavior is likely an artifact of the coverage area and survey timing:

> "However, the full extent of erosion during the comparably powerful 1997-98 event was probably not recorded due to two important factors. First, topographic survey coverage in 1997-98 wasn't as spatially extensive as in more recent years, with some of the sections of coastline anecdotally most impacted (e.g., California) having particularly poor or spotty coverage, or none at all. Second, the Lidar survey utilized to establish the post-El Niño shoreline for many of the California study sites wasn't collected until April 1998, when beaches were already rapidly recovering, aided by the greater availability of river-supplied sediments from the anomalously high rainfall that winter. This was not the case in 2015-16 for the watersheds adjacent to the California sites where rainfall was significantly below average compared to a typical winter. In recent years, surveys were more frequent throughout the year and/or were conducted during beach minima conditions in the winter."

The Section 'Historical significance of the 2015-16 El Niño' is largely a repeat of the previous two sections (especially the first paragraph). There is some 'new' information that would be better incorporated in the two previous sections.

The first sentence of this paragraph was deleted to avoid some unnecessary repetition among sections, and combined with the 2nd paragraph to make it a more efficient and focused section.

Lines 173-181 argue that the upper beach and dune response was quite limited during the 2015-16 winter because the beaches were in a relatively healthy state and widespread beach nourishment had taken place over the previous years. But what then explains the below average seasonal response to the stronger El Niño winter of 1997-1998?

See comments above.

Lines 183-198 are somewhat off-topic and rather speculative. It all depends on how important the fluvial sediment supply is for maintaining the beaches, and that really is a different topic/paper.

For some sections of the study area (e.g., Central California), this is critically important, and while a thorough evaluation of this factor is certainly a different paper, we felt it was an important topic to raise here. For example, per Barnard et al. (2010. Dramatic beach and nearshore morphological changes due to extreme flooding at a wave-dominated river mouth. *Marine Geology*, Volume 273 (1-2), p. 131-148, http://dx.doi.org/10.1016/j.margeo.2010.01.018), as much as ten years of littoral sediment supply can be delivered to the Central Coast during exceptional rainfall events, leading to 10's of meters of local shoreline accretion.

Lines 208-213 make reference to the sea-level anomaly and how this can be a proxy for sea-level rise. The sea-level anomaly for 1997-1998 far exceeded the 2015-2016 anomaly, but the coastal response during 1997-1998 was less than average. Again, this needs explanation.

Per our responses above, we have more thoroughly addressed the "moderate" behavior of 1997-98.

The beach change figure 4 contains some rather puzzling results that deserve explanation. Firstly, for several sites there is a significant beach widening trend over the last 20 years – it seems these beaches have significantly decreased their vulnerability. Secondly, the Camino Mallorca site shows the largest reduction in beach level occurred in the years before 2015-2016, directly contradicting the main conclusion of this paper. Thirdly, it is unclear what shoreline is plotted in this figure (MSL, MHW, MHHW).

1) The focus of this paper is on the seasonal response of beaches to anomalous forcing, not long-term coastal behavior. Trends are important to the long-term vulnerability of these coastal settings, but a thorough analysis of long-term trends is beyond the scope of this paper. That being said, we do directly address this topic in the discussion section:

    > "Although erosive conditions were clearly amplified in 2015-16, large landward shifts in shoreline positions did not translate to pervasively severe erosion of the dunes and bluffs that back the beaches in the Pacific Northwest. This is likely due to the fact that these beaches have generally been accreting since the previous 2009-10 El Niño, and were in a significantly prograded state in summer 2015 due to the previous mild winter. As a result, increased beach sand volumes moderated the landward erosion resulting from increased wave energy and water levels. Similarly, recent nourishments along several beaches in Southern

California also prevented shoreline retreat from reaching landward extremes during the winter of 2015-16, thereby providing more storm protection for dunes and adjacent coastal infrastructure[39]. Based on this recent behavior, such naturally or artificially sediment-rich coastal settings are likely to be more resilient to future storm impacts."

2) This is a drought-related phenomenon, which is why we highlighted the importance of considering the coastal sediment supply/watershed behavior in the context of the seasonal behavior, i.e., noting both the maximum seasonal excursions and beach minima. In the case of Camino Mallorca, and by proxy other parts of this study region, the beaches were already depleted due to reduced sediment supply, and shorelines therefore were pushed to historical minima when this large El Niño struck the coast in 2015-16, when relatively narrow beaches prevailed. We added the following text to further highlight this point:

    "This phenomena is clearly observed in the sand height time-series from Central California (Fig. 4, bottom), which shows a sharp decrease coincident with the onset of the drought in 2013, followed by record low sand heights in response to the 2015-16 El Niño event. Record low sand levels have persisted in this location through September 2016."

3) Added the following text to the figure caption: "For local shoreline proxy information see Supplementary Table 1."

Reviewer #2:
This is an important contribution that not only puts the significant of the 2015-16 ENSO event into a longer term historical perspective, but also provides important perspective on what the oceanographic conditions and shoreline responses that were experienced during this winter may mean for the future of shorelines/coastlines of the west coast states. The paper brings together very comprehensive data sets that span an important period of time but also a significantly large geographic area, which is unique relative to past shorter term or smaller areal descriptions or analyses. The quality of the date is high, the multiple authorship adds important breadth and geographic coverage, and the paper is a nice integration of both the forcing functions/physical processes and the shoreline responses.
Figures are nicely done, very understandable and present a great compilation of long term wave/water level/shoreline conditions that will be a valuable data source for other workers. Methods are straightforward and easy to understand and follow.

I am attaching a few specific comments, some other historic data and references that the authors may or may not be aware of. There have been data collected during previous ENSO winters (1983 and 1997-98) that do provide some added perspective on what was observed this 2015-16 winter, but that weren't referenced.

Lines 55- 56. "However, a detailed record of coupled oceanographic forcing (i.e., waves and water levels) and coastal response during these extreme events is limited primarily to anecdotal reports for the 1982-83 event." -There is one published graphic of the coincidence of predicted tidal levels and wave heights during the 1983 ENSO, which shows why this winter was so damaging to coastal California

The coincidence of high tides and storms is another important factor. We added a paragraph and citation (Flick 2016) to address this topic:

"The potential for even more extreme coastal erosion during the 2015-2016 El Niño was also moderated by the earlier onset of peak annual high tides and the seasonal water level anomaly associated with El Niño. The fall 2015 peaks in California, for example, were significantly earlier

than the winter peaks that occurred during the El Niños of 1982-83 and 1997-98, thereby reducing the probability for the coincident arrival of the largest waves and water levels of the 2015-16 event.[40]"

Line 67. I think convention is to hyphenate sea-level rise.

With increasing use the hyphen is becoming less common, but we will leave this to the discretion of the journal editor.

Line 98. Looking at NOAA water level anomalies, unless I am not understanding the use of "highest anomaly" for the 2015-16 water levels, the Monterey, California tide gage recorded an anomaly of 12.9 " or 32 cm anomaly (observed above predicted- although not listed as "verified" when I looked this up initially). See attached plot for January 5, 2016.

There were larger daily-scale anomalies at each of the water level stations in the study area throughout the winter due to low pressure systems and strong winds (i.e., storm surge) periodically affecting a given region, but here we focus on the seasonal averages.

Lines 122-127 These are good data, cover a broad geographic area and put the 2015-16 ENSO winter into recent historical perspective, in terms of coastal impact. There have been historical measurements from prior large ENSO winters but it's not clear from these plots that they are included. Do the comparisons include Kristen Brown's MA thesis (while she was working at USGS) on 1997-98 beach profile changes (pre-, mid and post-1997-98 ENSO- see attached summary) along central California coast, and John Dingler's.

These studies/data were not included as they lacked geodetic control, thereby eliminating comparison with more recently collected data, and are only found in the grey literature.

Lines 144-45- "including the most landward shoreline positions observed for the majority of beaches in California since topographic data collection began twenty years ago". What is important to keep in perspective, however, is that much of this 20 year period (~1999-2014) was a negative/cool PDO period such that large ENSO events wouldn't have been expected, and that extreme beach erosion or retreat wouldn't have been expected either, in contrast to the previous two decades (1978-1999), a warm/positive PDO cycle.

An excellent point, but by looking back to as early as 1871, this last El Niño still ranks in the top 3 regardless of PDO phase, and by proxy so would the expected oceanographic forcing and coastal response. Further, many shorelines reached historical minima, which further underlines the strength of 2015-16 in light of the possibility of it occurring during a cool PDO period, when shorelines would be expected to be more seaward/accretionary in general.

Line 168: sea-level rise

See above.

Lines 169-171- In addition to providing insight into possible future conditions when extreme El Niños are more frequent, the 2015-16 El Niño winter may have disrupted the dynamic equilibrium of many U.S. West Coast beaches for years to come…The beach surveys done by Kristen Brown as part of

her MA thesis with USGS showed severe erosion in 1997-98 along the central coast of California but then full recovery by the next fall.

Yes, and I think this validates our discussion of the importance of recovery in relation to the fluvial sediment supply/rainfall conditions of a given El Niño event. As we discuss, the rainfall in California in 2015-16 was abnormally low, which is likely related to the poor recovery from the proxy sand height time-series through fall 2016 in Central California (Fig. 4, bottom).

Lines 192-193: "Along with the historical trend of declining sediment supply, these 21st century climate projections would promote less runoff and reduced fluvial discharge rates, likely further reducing the coastal sediment supply." Somewhat surprisingly, despite the significant reductions in fluvial sediment supply due to construction of dams and debris basins in southern California, the beaches haven't uniformly or consistently shown reductions in width. (Orme, A.R., Griggs, G.B., Revell, D.L., Zoulas, J.G., Grandy, C.C., and Koo, J. 2011. Beach changes along the Southern California coast during the twentieth century: A comparison of natural and human forcing factors. Shore and Beach 79(4): 38-50)

A complicated topic worthy of further investigation, but certainly the Central California beaches we studied here, and largely the focus of the above paper (the geographic coverage is actually quite similar despite the title), show a steady decline over the last decade (green line in Figure 4, top). In many locations, the Southern California beaches do have this trend (i.e., lack of long-term erosion) due to nourishments, which prevented a few of our study site shorelines from reaching historical minima.

Reviewer #3:
This manuscript presents an overview of the coastal response along the US west coast, from southern California up to the Washington coast, to the 2015-16 El Nino event, which is identified as one of the strongest El Nino events in the historical record.

The study assesses the local physical drivers, sea level anomalies and wave climate, in context with measurements of shoreline position, to identify that the 15-16 erosional event lead to the most landward shoreline positions observed for the majority of the Californian coast since records began.
The study provides a very timely presentation of the coastal response to the recent El Nino event, and I would anticipate would be of interest to the readership of Nature Communications. I have a few comments below which I would recommend the authors consider before the paper is published.

I commend the authors on a nice study.

(abstract) The manuscript is set up describing the wide spread coastal response along the Pacific margin to El Nino events, but then limits analysis and later discussion to only the US west coast, and becomes quite US centric. Given the other work these authors have been involved in, it would be nice to see discussion of implications more broadly, increasing the interest to a much broader readership.

We modified the following sentence in the abstract to more clearly convey that the West Coast is a good proxy for ENSO impacts across the Pacific:

> "Further, some climate change projections suggest a possible increase in the frequency of extreme El Niño and La Niña events, **which would affect coastal communities across the entire**

Pacific Basin margin, making it critical to document the forcing and response of historically strong events as a possible proxy for future coastal vulnerability."

In the introduction we do demonstrate that the scope of both El Niño and La Niña impacts is not limited to the U.S. West Coast. For example:

"The region experienced substantial increases in coastal hazards during previous El Niño winters, and has been shown to broadly represent conditions across the Central and Eastern North Pacific[5,15]."

We also conclude the paper with following line:

"Therefore, the 2015-16 El Niño also provides an indication of future background coastal water level conditions and the associated beach hazards that will become more common during typical winters. The added potential for severe flooding and erosion will be compounded during El Niño winters with extreme wave energy and seasonally-elevated water levels, posing increasing threats to coastal populations across the U.S. West Coast and beyond."

(line 38), gives a figure of US$11.5 billion damages associated with the 82-83 El Nino. Using this figure, which relates to all El Nino damages – a lot of which are associated with industries well away from the coastal fringe (agriculture, or fisheries), is a bit misleading. Is there a figure available on what the damages associated with coastal erosion/shoreline position/flooding are? This would be a more relevant figure to quote.

We haven't been able to track down any data that parses out the damages specific to the coast except at local/regional scales. The point here is to convey the global importance of the event.

(line 79) Referring to the 95th percentile as extreme is not appropriate. Hourly data is used, and the 95th percentile corresponds to an average of every 20th measurement (more than once per day) is greater than this value. Some other method of characterizing 'extreme' waves should be considered.

The 95% exceedance represents just 4.5 days (108 hours) of a winter storm season (total of 2160 hours), or just several storm events, which we would argue is a reasonable approximation of extreme waves that drive coastal response. However, to better satisfy varying definitions of extreme waves, we have added columns in Supplementary Table 2 for the 98, 99,99.5 and 99.9% exceedance values, and alluded to them in the main text and the Figure 3 caption, noting that these other metrics yield similar results, e.g.,:

"note the top 0.1, 0.5, 1 and 2% of wave energy flux is also included in this table, yielding an even greater discrepancy in extreme wave energy flux for the winter of 2015-16 compared to all other winters on record. In the text hereafter, however, we refer exclusively to the top 5% as 'extreme'."

(line 79-81) sentence needs editing.

Done

(line 95). The wave conditions during the 15/16 El Nino are identified as being atypical relative to prior el nino events.

True, in terms of the wave direction. This is discussed quite extensively, with some updated text to ensure this point is clear.

On lines 157-162, the authors attribute these differences to background trends in wave climate. The analysis of this is somewhat superficial and I think the paper would be substantially strengthened by a more rigorous analysis of this. The counter argument is that by being atypical, they provide a poor model of what may occur during future El Nino events, as is argued in other sections of the manuscript.

A thorough analysis of storm tracks and their long-term migration patterns is far beyond the scope of this paper, although the clear northerly shift in wave approach between the last two major El Niños is well-founded and discussed, and the peer-reviewed literature cited here indicates that this is likely related to a longer term trend. Therefore the impacts of future El Niños are more likely to be focused further north along the West Coast, and likely will be more similar to impacts seen in 2015-16 than in 1982-83 and 1997-98.

(line 143 – and elsewhere in the manuscript). Reference to wave energy, and wave power (wave energy flux) appears to be used interchangeably without consideration through the manuscript. This should be tightened up.

Done.

(line 150-151). The wording of 'strong latitudinal variations' doesn't capture what I think the authors are trying to convey – being the differences in latitude of the storms between the two events. As worded, it suggests that during each event, there is similar variations in latitude of the storms. Some editing of the text needed.

Modified this sentence to read:

> "The primary difference in wave energy flux between the most powerful El Niño events of the last two decades (i.e., 1997-98 and 2015-16) appears related to a latitudinal shift in the primary storm tracks and resulting wave generation location."

Figure 3, and discussion around line 210. It is not clear whether the positive sea level anomalies along the US west coast are a common signal of all El Nino events, or whether the authors are just proposing this positive anomaly provides some indication of a possible future sea-level rise signal. I think the point is lost that the positive anomalies will on top of the background SLR in future, exacerbating potential effects.

Modified the last sentence to clarify this point:

> "Therefore, the 2015-16 El Niño also provides an indication of future background coastal water level conditions and the associated beach hazards that will become more common during typical winters. The added potential for severe flooding and erosion will be compounded during El Niño winters with extreme wave energy and seasonally-elevated water levels, posing increasing threats to coastal populations across the U.S. West Coast and beyond."

Reviewers' comments:

Reviewer #1 (Remarks to the Author):
None - I am happy with the changes to the manuscript following my review

Reviewer #2 (Remarks to the Author):

The authors have done a very complete job of responding to each reviewer's comments and I am happy to provide my sign off for acceptance and publication

Reviewer #3 (Remarks to the Author):

Review of manuscript submitted to Nature Comms
Barnard et al
This is a review of the revised version of this manuscript, following some minor changes by the authors. The authors have rewritten various passages of text, and included some new references, but no further analysis has been carried out for the revision.
While some aspects of my prior comments have been addressed, several haven't, and in some cases the revised version is contrary to my recommendations (and seemingly not due to contrary reviewer comments). For example, my previous review outlined my concern that the paper misused the term 'extreme' by looking at just the 95th percentiles. Not only have the authors largely ignored the comment in their study (by continuing use of 95th percentile as their statistic to represent storm wave conditions – I acknowledge they have added higher percentile values to supp. Table 2), they have increased the use of the term 'extreme' conditions, by including it in the title. I think this is inappropriate. By the authors admission, they are talking about conditions which occur for 4.5 days in a winter season. These are typical winter storm conditions (probably accounting for 2-3 storms), as opposed to extreme conditions, and the title and presentation of results should reflect this. I recommend 'extreme' be removed from the title, and other language be used through the body of manuscript.
Another point which I don't think has been addressed adequately is the comments regarding trends in storm track conditions and their influence on wave climate conditions on the western North American coast. The discussion around trending wave height increases further north along this coast is contrary to the historical trends along this coast (e.g., Gemmrich et al., 2011, who showed no statistically significant historical trends in storm wave heights along this coast, with a tendency for positive trends in the south and negative trends in the north).
Gemmrich et al. (2011) Observational changes and trends in northeast Pacific wave records. GRL, doi:10.1029/2011GL049518
Some other minor comments:
I note in the revised version the authors have altered language from historical to observed historical. I am not sure this improves the distinction which is the intended purpose, being measured or quantified – perhaps this can be clarified more clearly.
The authors respond that the quoted $figure conveys the importance of the 2015-16 el-nino. There is an inference that some component of this is related to coastal hazards, and this is not clear. The major proportion of this $ figure is related to resources and its misleading to point to this to justify the coastal hazards study without some clarification.
Line 155-159. Long sentence needs rewrite.
Line 213-217. Long sentence needs rewrite.

Response to Reviewer Comments

Reviewer #1:
None - I am happy with the changes to the manuscript following my review.

Thank you so much for your time and expertise in re-reviewing this manuscript.

Reviewer #2:
The authors have done a very complete job of responding to each reviewer's comments and I am happy to provide my sign off for acceptance and publication.

Thank you so much for your time and expertise in re-reviewing this manuscript.

Reviewer #3:
This is a review of the revised version of this manuscript, following some minor changes by the authors. The authors have rewritten various passages of text, and included some new references, but no further analysis has been carried out for the revision. While some aspects of my prior comments have been addressed, several haven't, and in some cases the revised version is contrary to my recommendations (and seemingly not due to contrary reviewer comments).

We spent considerable effort responding to each of the reviewer's concerns the first time, spending quite a number of pages responding to each item in careful detail with strong, reasoned arguments, explicit modifications to the text, and additional data analyses (more on this below).

For example, my previous review outlined my concern that the paper misused the term 'extreme' by looking at just the 95th percentiles. Not only have the authors largely ignored the comment in their study (by continuing use of 95th percentile as their statistic to represent storm wave conditions – I acknowledge they have added higher percentile values to supp. Table 2), they have increased the use of the term 'extreme' conditions, by including it in the title. I think this is inappropriate. By the authors admission, they are talking about conditions which occur for 4.5 days in a winter season. These are typical winter storm conditions (probably accounting for 2-3 storms), as opposed to extreme conditions, and the title and presentation of results should reflect this. I recommend 'extreme' be removed from the title, and other language be used through the body of manuscript.

The reviewer is focused on our use of the word "extreme" to refer to the largest of winter waves, which in their view represents "typical winter storm conditions". While "typical winter storm conditions" certainly include a small fraction of unusually large waves relative to that winter's mean wave height, it is exactly this fraction that is of interest, as it is the "extreme" (i.e., largest) waves in any given winter that cause most of the shoreline erosion (see below for discussion of our quantitative/statistical definition of the term, and citations of numerous papers in the field that also use this definition of "extreme"). Thus we feel that it both accurate and appropriate to use "extreme" to refer the largest waves in a given winter, and to use it in the title, as it is both the occurrence of "extreme" waves in any given winter, and <u>increases in the magnitude of</u> "extreme" waves (i.e., "extreme extremes") during El Niño that affect shoreline erosion, which is the central premise of this paper. The reviewer also suggests that we ignored his request to explore other metrics. In fact, we ran a suite of other metrics (top 0.1, 0.5, 1 and 2% of wave energy flux) that could be defined as "extreme", we clearly noted in the text that

they were consistent with the top 5% we are defining as ‘extreme’ in this paper, and we added all the new data to the supplementary section. In the latest revision, we modified the text to further clarify that the results of the top 5% “extreme” metric are consistent with the additional metrics:

> “note the top 0.1, 0.5, 1 and 2% of wave energy flux is also included in this table, **yielding results consistent with the top 5% metric**, but with an even greater discrepancy in extreme wave energy flux for the winter of 2015-16 compared to all other winters on record.”

For our wave energy discussion, “extreme” merely refers to the fine, maximum value tail of a PDF, which is variably defined in the literature for waves (e.g., heights, energy flux, power, etc.) as varying from the ~top 10% (i.e., 90% exceedance, e.g., Bromirski et al., 2013) to the top 0.5% (e.g., Ruggiero et al., 2010). Extreme wave energy is more commonly defined by a relatively wider fraction of the pdf (e.g., top 5%) compared to water levels, as “extreme” wave energy from multiple storms is more tightly linked to geomorphic change (i.e., coastal erosion), which is discussed in Allan and Komar (2006 [they looked at the peaks of 5 distinct storms, for example, among other metrics]), and which we also explored at length in Barnard et al. (2012; 2015), where we found that the top 5% of wave energy flux showed the best statistical relationship with measured seasonal coastal response. Other prominent researchers in this field and along this coast have used similar metrics (e.g., Menendez et al., 2008 [top 2%]; Graham et al., 2013 [top 1% and 10%]; Erikson et al., 2015 [top 1% and 5%]). The benchmark for extreme wave-run up has long been reported as the top 2% (Stockdon et al., 2006), and flood risk is more typically associated with water levels at the very end of the tail (e.g., top 0.01%), hence “extreme” for water levels may be shifted more to the right along a PDF, but such a measure of “extreme” is less commonly applied to waves per our reasoning above. Regardless, there is no accepted single quantitative definition of “extreme” in the literature, so we have chosen one to describe a metric for wave energy flux that represents conditions during the largest storms for each winter, which is also strongly correlated to the measured seasonal coastal response. While it seems that the reviewer would be happier with an extreme metric that reflects just a few hours of wave measurements, we would argue that such a small interval could restrict data to waves from a single anomalously large storm (or even a few hours at the peak of the largest storm), which could happen virtually any winter, but that looking at the top 5% (or something similar) reflects the extreme conditions for the largest series of storms that struck that winter, and thus is more likely reflective of the dominant mode of climate variability, oceanographic forcing conditions, and ultimately coastal response for that winter. In support of this, Allan and Komar (2006) found that the scatter in regression plots of historical wave height trends progressively increased with the increasing consideration of fewer wave height measurements in their definition of “extreme”, that is further toward the maximum tail of the PDF.

Finally, the use of “extreme” in the title is meant to convey the fact that the waves and coastal response were highly anomalous - we changed this from "historically significant" which reviewer #1 didn't like. The winter of 2015-16 brought the largest waves ever recorded to the coast as well as the most severe erosion, so calling that “extreme” seems both accurate and appropriate.

Another point which I don’t think has been addressed adequately is the comments regarding trends in storm track conditions and their influence on wave climate conditions on the western North American coast. The discussion around trending wave height increases further north along this coast is contrary to the historical trends along this coast (e.g., Gemmrich et al., 2011, who showed no statistically significant historical trends in storm wave heights along this coast, with a tendency for positive trends in the south and negative trends in the north).

Gemmrich et al. (2011) Observational changes and trends in northeast Pacific wave records. GRL, doi:10.1029/2011GL049518

Gemmrich et al. (2011) actually does show an INCREASE in waves for the buoys from extreme far northern California to Washington, which is adjacent to the northern part of our study area (i.e., our Oregon and Washington study regions), and a slight decrease for the buoys in British Columbia and southern AK, which are NOT in our study area. Gemmrich (2011) does not include any buoys adjacent to our 4 study regions in California. Thus, closer examination of the study the reviewer cites shows that it actually SUPPORTS our assertion that waves are getting bigger off the coast of OR and WA due to the northern migration of storm tracks. In our paper we simply state that the extreme wave conditions during 2015-16 were more pronounced in the northern half of our study area (namely OR/WA) and less so for CA relative to 1997-98 (and anecdotally for 1982-83), which is consistent with a progressively poleward shift in storm tracks. This increase in wave height trends moving north along the U.S. West Coast (i.e., for the Pacific Northwest in comparison to California, where the trend is effectively flat) over the last several decades is also clearly noted in a series of other publications, including Allan and Komar (2006), Menendez et al., (2008, also shows a decrease off the coast of Canada similar to Gemmrich), and Ruggiero et al. (2010). Further, this continued trend is also noted in the projected increase in wave heights for the British Columbian coast over the 21 century due to an increased northern shift of storm tracks, as suggested by Graham et al. (2013) and Erikson et al. (2015), with a correlative decrease along much of the U.S. west coast to the south. All these studies are perfectly in line with our hypothesis that storm tracks have moved and may continue to move further poleward during successive El Niño events, with the zone of increasing wave heights moving further north through time as well.

We have added the aforementioned references to the manuscript to further bolster this hypothesis (several were in there already) and clarified our reasoning, along with editing/adding the following text in the discussion section:

> "A northerly shift in storm tracks during the El Niño of 1997-98 compared to 1982-83 is suggested by precipitation records across California[32] as well as reports of significant flooding and coastal erosion[13,33], indicating more pronounced impacts from local storms in Southern California during the 1982-83 winter relative to 1997-98. This evidence of a progressive northern migration of storm tracks during El Niño winters along the U.S. west coast is consistent with the observed multi-decadal trend of poleward Hadley cell expansion and, therefore, the location of the sub-tropical jet stream[34]. Measured multi-decadal increases in wave heights for the Pacific Northwest relative to California [4,35-37] is evidence of this broader trend, as is the predicted poleward migration of storm tracks and correlative northerly shift in the focus of extreme wave impacts along the west coast of North America noted in global wave modeling projections for the 21st century[38,39]."

An even more in-depth investigation of storm tracks is another paper entirely, and begins to get off topic from the focus of this investigation.

Some other minor comments:
I note in the revised version the authors have altered language from historical to observed historical. I am not sure this improves the distinction which is the intended purpose, being measured or quantified – perhaps this can be clarified more clearly.

The differences (or lack thereof) between "observed" and "measured" could be considered a matter of editorial opinion. Nevertheless, in the interest of achieving the utmost clarity we have in most instances inserted "measured" in place of "observed" per the reviewer's suggestion.

The authors respond that the quoted $figure conveys the importance of the 2015-16 el-nino. There is an inference that some component of this is related to coastal hazards, and this is not clear. The major proportion of this $ figure is related to resources and its misleading to point to this to justify the coastal hazards study without some clarification.

Added the phrase "…including significant losses along the coast".

Line 155-159. Long sentence needs rewrite.

Re-written and broken up into two sentences.

Line 213-217. Long sentence needs rewrite.

Broken up into two sentences.

References cited above:

Allan, J. C., & Komar, P. D. Climate controls on US West Coast erosion processes. *J. Coast. Res.* **22,** 511–529 (2006).

Barnard, P. L. *et al.* The impact of the 2009-10 El Niño Modoki on U.S. West Coast beaches. *Geophys. Res. Lett.* **38,** L13604 (2011).

Barnard, P. L. *et al.* Coastal vulnerability across the Pacific dominated by El Niño/Southern Oscillation. *Nature Geosci.* **8,** 801–807 (2015).

Bromirski, P. D., D. R. Cayan, J. Helly, and P. Wittmann (2013), Wave power variability and trends across the North
Pacific, J. Geophys. Res. Oceans, 118, 6329–6348, doi:10.1002/2013JC009189.

Erikson, L.H., Hegermiller, C.A., Barnard, P.L., Ruggiero, P. and van Ormondt, M., 2015. Projected wave conditions in the Eastern North Pacific under the influence of two CMIP5 climate scenarios. *Ocean Modeling*, Volume 96, p. 171-185, http://dx.doi.org/10.1016/j.ocemod.2015.07.004

Gemmrich, J., Thomas, B., Bouchard, R., 2011. Observational changes and trends in northeast Pacific wave records. Geophys. Res. Lett. 38, L22601.

Graham, N.E., Cayan, D.R., Bromirski,P.D., Flick, R.E., 2013. Multi-model projections of twenty-first century North Pacific winter wave climate under the IPCCA2 scenario. Clim. Dyn. 40, 1335–1360.

Menéndez, M., Méndez, F.J., Losada, I.J., Graham, N.E., 2008. Variability of extreme wave heights in the northeast Pacific Ocean based on buoy measurements. Geophys. Res. Lett. 35, L22607.

Ruggiero, P., Komar, P.D., Allan, J.C., 2010. Increasing wave heights and extreme value projections: the wave climate of the US Pacific Northwest. Coast. Eng. 57, 539–552.

Stockdon, H. F., Holman, R. A., Howd, P. A., and Sallenger, A. H., 2006. Empirical parameterization of setup, swash, and runup. Coastal Eng. 53(7), 573–588.

REVIEWERS' COMMENTS:

Reviewer #2 (Remarks to the Author):

I am satisfied with the authors comments to all reviews.

Reviewer #3 (Remarks to the Author):

This is a response to the authors comments provided to a prior review of the manuscript. Changes to the previous version of the manuscript in response to my prior comments are minimal. I support the changes that have been made.

The main objection is to the reference of 95th percentile representing ‘extreme’ conditions. This is largely a nomenclature or definition issue. The authors have cited a number of studies to justify their choice of the 95th percentile to represent extremes. However in many instances, the cited studies do not support the choice of a 95th percentile to represent extremes.

For example, the authors refer to Bromirski et al as defining extremes on the basis of the top 10% (use the 90% exceedance). Bromirski et al. 2013 do not define extremes based on the 90th percentile, but use a threshold of the 90th percentile, and look at events that exceed the 90th percentile for at least 12h. This results in quite different statistics (much more extreme conditions) than simply reporting the 90th percentile.

A similar peaks over threshold approach is taken by Menendez et al. (2008) cited by the authors. Mendendez et al use a threshold of the 95th percentile, and look at the peak of these events and fit a statistical extreme model to the distribution of events. Again, this provides a dataset with more extreme conditions than simply using the 95th percentile.

Other studies use different language, e.g., Graham et al 2013 present similar variables under the language of winter wave climate – this is more appropriate than describing as extremes.

My issue is that interest in extreme conditions is for design of coastal and offshore infrastructure, or integrity of coastal systems impacted by rare events. If designing a system to have a 30-yr life, one wants to know what these rare ‘extreme’ conditions are that only occur with such low frequency . Biasing results with low values (e.g., several typical winter storms) presents a risk to that design. The use of the term makes it more difficult to isolate the studies which are focussed on ‘extreme’ conditions.

Certainly the event in question was exceptional and an extreme event, so by that argument the use of ‘extreme’ in the title is probably appropriate. I maintain that using the 95th percentile as representative of extreme wave conditions is not, and recommend (again) that either the language be modified, or an alternative statistic be used. I do not wish to see this manuscript again, so happy for the editor to use their judgement.

Response to Reviewer Comments

Comments in italics
Responses in standard case.

Reviewer #2:
I am satisfied with the authors comments to all reviews.

Thank you so much for your time and expertise in reviewing this manuscript again.

Reviewer #3:
This is a response to the authors comments provided to a prior review of the manuscript. Changes to the previous version of the manuscript in response to my prior comments are minimal. I support the changes that have been made.

The main objection is to the reference of 95th percentile representing 'extreme' conditions. This is largely a nomenclature or definition issue. The authors have cited a number of studies to justify their choice of the 95th percentile to represent extremes. However in many instances, the cited studies do not support the choice of a 95th percentile to represent extremes.

For example, the authors refer to Bromirski et al as defining extremes on the basis of the top 10% (use the 90% exceedance). Bromirski et al. 2013 do not define extremes based on the 90th percentile, but use a threshold of the 90th percentile, and look at events that exceed the 90th percentile for at least 12h. This results in quite different statistics (much more extreme conditions) than simply reporting the 90th percentile.

A similar peaks over threshold approach is taken by Menendez et al. (2008) cited by the authors. Mendendez et al use a threshold of the 95th percentile, and look at the peak of these events and fit a statistical extreme model to the distribution of events. Again, this provides a dataset with more extreme conditions than simply using the 95th percentile.

Other studies use different language, e.g., Graham et al 2013 present similar variables under the language of winter wave climate – this is more appropriate than describing as extremes.

My issue is that interest in extreme conditions is for design of coastal and offshore infrastructure, or integrity of coastal systems impacted by rare events. If designing a system to have a 30-yr life, one wants to know what these rare 'extreme' conditions are that only occur with such low frequency . Biasing results with low values (e.g., several typical winter storms) presents a risk to that design. The use of the term makes it more difficult to isolate the studies which are focussed on 'extreme' conditions.

Certainly the event in question was exceptional and an extreme event, so by that argument the use of 'extreme' in the title is probably appropriate. I maintain that using the 95th percentile as representative of extreme wave conditions is not, and recommend (again) that either the language be modified, or an alternative statistic be used. I do not wish to see this manuscript again, so happy for the editor to use their judgement.

We appreciate the reviewer more explicitly explaining the objection to the term “extreme”, and have modified that language accordingly to refer to “extreme” wave conditions (i.e., top 5%) simply as “elevated”.
